# The scent of serenity: lessons learned from olfactory-enhanced virtual reality for stress reduction in isolated and confined environments

**DOI:** 10.3389/fpsyg.2026.1769314

**Published:** 2026-07-01

**Authors:** Renee Abbott, Dale W. Russell, Suzanne T. Bell, Ana Diaz-Artiles

**Affiliations:** 1 Department of Aerospace Engineering, Texas A&M University, College Station, TX, United States; 2 Department of Psychiatry, Uniformed Services University of the Health Sciences, Bethesda, MD, United States; 3 Behavioral Health and Performance Laboratory, Division of Biomedical Research and Environmental, NASA, Houston, TX, United States

**Keywords:** behavioral health intervention, cognition, immersive environments, multisensory integration, presence, restorative environments, virtual nature

## Abstract

**Introduction:**

Chronic stress and the lack of restorative environments negatively impact behavioral health, especially in isolated, confined, or operational settings. Virtual reality (VR) is a promising behavioral health tool, with virtual nature scenes shown to promote relaxation and improve mood. Enhancing VR with olfactory stimuli to create a multisensory virtual nature experience may amplify these restorative effects.

**Methods:**

This exploratory study examined the feasibility of utilizing an olfactory-enhanced VR (OVR) intervention, comparing psychological and cognitive performance outcomes over 2 weeks aboard a US Navy ship for three groups: OVR, standard VR, and a no-intervention control. Twenty-nine participants were assigned to the Control, VR only, or OVR group. Participants completed cognitive performance tasks and surveys measuring affect, perceived stress, presence, and restoration.

**Results:**

The OVR group showed significant reductions in negative affect immediately post-intervention (*p =* 0.02, d = −1.3) compared to baseline. Both VR and OVR groups rated Total presence *low* (below the 50th percentile), but General Presence, Spatial Presence, and restorative qualities of the virtual environments were rated highly. Cognitive performance remained generally stable but revealed potential decrements in sustained vigilance and emotion identification as well as a decline in risk-taking propensity, though effects were small and inconsistent across cognitive tasks. Qualitative feedback provided preliminary evidence of acceptability and perceived benefits. However, results should be interpreted cautiously, as the study was exploratory and not powered for robust inference across the large number of assessed outcomes.

**Discussion:**

Overall, olfactory-enhanced VR was associated with immediate improvements in negative affect but lacked evidence of extended psychological or cognitive performance benefits under the conditions tested. These findings provide preliminary feasibility evidence supporting further investigations examining multisensory VR as an acute affective-support tool in operational environments. Further research should explore benefit duration, exposure dosage, and individual differences to optimize the effectiveness of VR nature interventions.

## 1 Introduction

Chronic stress is a pervasive public health concern with well-documented consequences for psychological functioning and behavioral health. These effects are especially salient in isolated, confined, and extreme (ICE) environments, such as those experienced by astronauts and military personnel, where additional stressors (e.g., physical and social isolation, monotony, and unpredictability) further compound the risk of psychological deterioration and cognitive decline (Bartone et al., 1998; Kanas et al., 2009; Kavanagh, 2005). Reduced sensory stimulation and sensory monotony in ICE environments are additional contributors to adverse behavioral health outcomes (Vessel and Russo, 2015), posing substantial risks in high-demand, high-stakes environments where sustained cognitive performance is essential.

Naval personnel, in particular, face environmental conditions that intensify psychological strain, including persistent noise, fluctuating temperatures, restricted diets, lack of privacy, limited natural light, and suboptimal sleep environments (Biggs et al., 2024; Biggs and Russell, 2023; Brooks and Greenberg, 2022; Jameson et al., 2023; Mallett et al., 2023; Schmied et al., 2024; Viboch et al., 2022). Such environmental constraints disrupt circadian rhythms and reduce opportunities for restorative experiences, thereby increasing susceptibility to mental health difficulties. Nearly 40% of enlisted sailors report experiencing severe or extreme stress during deployments (Mongilio, 2024), and Navy personnel report the highest rates of serious psychological distress across the branches of the US Armed Forces (Meadows et al., 2021). This underscores the urgent need for scalable, context-appropriate interventions that can mitigate the psychological toll of operating in austere conditions.

Exposure to natural environments (e.g., forests) is a well-established mechanism for promoting well-being and restoring depleted mental resources, as described in Attention Restoration Theory (Kaplan and Kaplan, 1989) and Stress Recovery Theory (Ulrich, 1983; Ulrich et al., 1991). Empirical evidence demonstrates that nature exposure improves mood, attention, and working memory, reduces burnout, increases life satisfaction, and supports stress recovery (Berman et al., 2008; Hansmann et al., 2007; Hartig et al., 2003; Mantler and Logan, 2015). However, access to nature is inherently limited in ICE environments.

Virtual reality (VR) is a portable and flexible platform for providing access to natural environments. VR-based nature exposure reliably reduces stress and anxiety and promotes relaxation in laboratory, medical, and operational settings as well as ICE environments (Anderson et al., 2017, 2023; Browning et al., 2020; Liszio et al., 2018; Nijland et al., 2021; Stetz et al., 2011). While prior efforts have centered on visual and auditory modalities, adding other sensory inputs could increase realism and immersion and enhance the psychological benefits of virtual nature (Mayer et al., 2009).

Olfaction in particular remains underutilized in VR applications due to technical challenges in odor generation and controlled delivery. The olfactory system plays a distinct role in emotion regulation due to its common neural substrates with brain regions associated with emotional processing such as the amygdala and hippocampus (Soudry et al., 2011). This allows odors to uniquely modulate affective state. Pleasant scents can elevate mood, reduce symptoms of depression, and facilitate psychological relaxation (Canbeyli, 2021; Kadohisa, 2013; Lehrner et al., 2000, 2005). Furthermore, olfactory stimuli can influence cognitive performance by enhancing vigilance, memory, and alertness, and modulating pain perception (Marchand and Arsenault, 2002; Moss et al., 2008; Norrish and Dwyer, 2005; Warm et al., 1991). Although the precise mechanisms remain under investigation, it is well-established that odors can modulate emotional states, and emotions, in turn, exert a measurable influence on cognitive functioning (Brosch et al., 2013; Broughan, 2002; Chepenik et al., 2007; Tyng et al., 2017). These studies underscore the potential value of incorporating olfactory cues into VR interventions designed to promote emotional regulation and psychological resilience.

Given the capacity of olfactory stimuli to alter psychophysiological states, their integration into VR environments presents a promising avenue for enhancing the efficacy of immersive interventions aimed at stress reduction and mood regulation. Thus, olfactory-enhanced VR may provide a neurobehavioral bridge between emotional regulation and cognitive performance, a critical consideration for sustained operations in ICE contexts. Previous work has shown that the incorporation of olfactory stimuli into relaxing, nature-based VR environments significantly enhances mood and reduces anxiety to a greater extent than audiovisual VR alone (Abbott and Diaz-Artiles, 2022). However, existing investigations have primarily focused on the short-term emotional effects of olfactory-enhanced VR, leaving open the question of whether these benefits persist and whether benefits may extend to the domain of cognitive performance.

To address this gap, the present study examined the effect of repeated exposure to relaxing VR environments with and without olfactory augmentation on psychological recovery and cognitive performance over a two-week period. Participants were assigned to a control group, a standard audiovisual VR group, or an olfactory-enhanced VR (OVR) group. The intervention was conducted in an operational setting, characterized by restricted access to natural environments.

The primary objective was to evaluate the impact of VR exposure on affect, presence, restorativeness, stress, and cognitive performance, and to determine whether the addition of olfactory stimuli enhances these effects. It was hypothesized that:

*H1*: the audiovisual VR and OVR interventions will produce immediate improvements in affect compared to baseline, with greater improvements in the OVR group;

*H2*: presence and perceived restorativeness in the virtual environment will be greater for the OVR intervention than the audiovisual VR intervention;

*H3a*: repeated exposure to the audiovisual VR and OVR intervention will lead to sustained improvements in affect and reductions in perceived stress over time compared to the control condition, with the strongest effects observed in the OVR group; and.

*H3b*: repeated exposure to the audiovisual VR and OVR intervention will lead to sustained improvements in cognitive performance over time compared to the control condition, with the strongest effects observed in the OVR group.

## 2 Methods

### 2.1 Study design and setting

This study employed a three-group, parallel design to evaluate the effects of repeated exposure to VR nature environments, with and without olfactory augmentation, on psychological and cognitive outcomes over a two-week period. This study was conducted during a two-week underway period aboard a U. S. Navy warship, where access to natural environments is restricted. This research complied with the tenets of the Declaration of Helsinki and was approved by the Institutional Review Board at Texas A&M University and the Naval Health Research Center. Informed consent was obtained from each participant.

### 2.2 Participants

Participants were recruited from the active-duty personnel aboard the warship during the underway period. To be considered for inclusion, participants had to be 18–64 years old, in good general health, and not lost all or part of their sense of smell. All participants completed a pre-screening questionnaire prior to enrollment. Twenty-nine active-duty U. S. Navy personnel (4 female; age: 31.4 ± 4.4 years) were recruited. Six participants did not complete the full protocol due to operational demands. This attrition resulted in unequal group sizes, which may have reduced statistical power for between-group comparisons.

### 2.3 Group allocation

Participants were assigned to one of three groups: control (no VR), audiovisual VR, or olfactory-enhanced VR (OVR). Participants were assigned using block randomization (block size = 6) to maintain balance across groups. Of the participants who completed the full protocol, 10 were in the control group, 5 in the VR group, and 8 in the OVR group.

### 2.4 Intervention

The nature VR intervention was designed to provide brief, immersive experiences in restorative environments, based on Attention Restoration Theory and evidence that exposure to natural environments can provide psychological and cognitive benefits. Participants assigned to the VR and OVR groups completed four VR sessions over the study period, while the control group received no intervention. Each VR session lasted approximately 30 min, including setup, 15 min of VR exposure, and post-session assessments. Participants freely explored the VR scene of their choice for 15 min. Immediately after VR exposure, participants completed the Positive and Negative Affect Schedule (PANAS), iGroup Presence Questionnaire (IPQ), and Perceived Restorativeness Scale (PRS), and reported VR sickness symptoms and optional feedback. VR sessions were intended to occur on separate days; however, due to operational constraints, there were three instances where two VR sessions were completed on the same day.

Participants were allowed to select any scene for each session to better reflect real-world operational use. Participants were generally expected to remain within a single environment during a session, although some elected to explore multiple environments. This also enabled individualized experiences, as both scene selection and interaction patterns influence sensory exposure (including olfactory stimuli in the OVR condition). All sessions were conducted in a designated space within the ship’s medical ICU and were supervised by a trained research assistant. The assistant provided instructions on equipment use, ensured participant safety, and addressed technical issues as needed.

**Figure 1:**
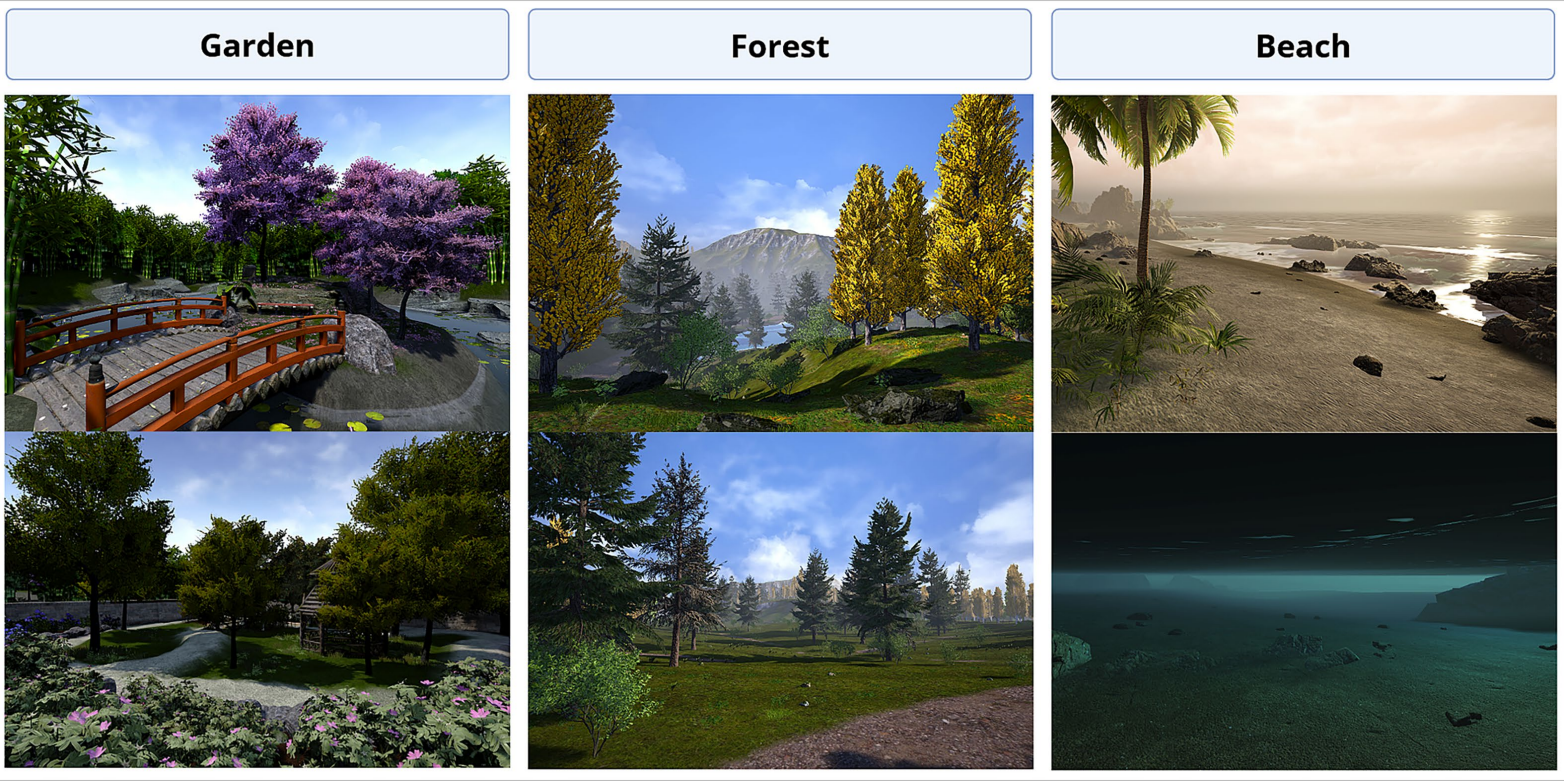
Representative screen shots of the relaxing nature VR environments with olfactory stimuli. The Garden contained fresh grass, rosemary, roses, blossom, and wet ground scents, the Forest had fresh grass, lavender, pine, wet ground and woods scents, and the Beach had beach, fresh grass, and wet ground scents.

*VR environments*: three computer-generated environments were custom developed using Unreal Engine 5: a garden, a forest, and a beach (Figure 1). Additional videos of the three VR scenes may be found at: https://youtu.be/7tHSgggd-Hk?si=Gv6VD14Gn_cn4wQS. Computer-generated environments were selected over pre-recorded HD or 360° videos to enable active navigation, as passive viewing has been associated with increased VR sickness (Chang et al., 2020). The environments were selected to represent a range of natural settings previously associated with psychological restoration. The garden environment combined built and natural elements and featured high plant biodiversity, which has been linked to enhanced restorative outcomes (Carrus et al., 2015). The forest environment included mountainous terrain and mixed vegetation and reflects commonly studied restorative settings, including those used in forest bathing (Shinrin-yoku) research (Wen et al., 2019). The beach environment included coastal features with rock formations and tropical vegetation, consistent with evidence that aquatic elements are associated with increased restorative effects (White et al., 2010).

All environments included both green and blue elements (e.g., ponds or ocean features) to support restorative potential. Scenes consisted primarily of natural elements with minimal built structures and did not include humans or animals. Each environment also contained interactive, physics-based objects (garden: stones, sunflowers; forest: pinecones, mushrooms; beach: starfish, coconuts), allowing users to engage actively with the environment.

*Equipment and interaction*: participants used an HTC Vive Pro Eye headset (HTC Corporation, Xindian, New Taipei City, Taiwan) with a wired connection to a Dell Precision (CPU Intel® Core™ i7-9850H @ 2.60 GHz, 32 GB RAM, NVIDIA Quadro RTX 4000 GPU). Navigation was enabled through handheld controllers, with both continuous locomotion and “point-and-teleport” movement available. Teleportation was included to reduce VR sickness risk (LaViola et al., 2017; Riecke and Zielasko, 2021).

*Audio*: audio was delivered through integrated on-ear headphones. Participants could adjust the volume as desired. Procedural, 3D audio that changed with the environment was used to enhance realism and reduce repetition (Kern and Ellermeier, 2020). The audio design consisted of three components:

1. a continuously looping base ambience track with (e.g., wind, rustling leaves, bird songs), with
2. location-specific sounds (e.g., waves near the ocean), and
3. randomized, transient “stingers” such as short bird calls or insect chirps, triggered at randomized intervals (15–40 s) with variation in pitch, source angle, and distance to mimic real world variation.

Acoustic properties of each scene were quantified using root mean square (RMS) energy, representing overall signal intensity, and band energy ratios derived from 1-min representative audio clips. Frequency bands were defined as low (0–250 Hz), mid (250–4,000 Hz), and high (4000–8,000 Hz), with values reported in Table 1.

**Table 1:** Descriptions and attributes of the three computer-generated VR scenes.

VR Scene	Garden	Forest	Beach
Description	A small bamboo garden on a sunny day featuring a variety of vegetation (e.g., juniper, Japanese maple, and elm trees, rosemary, ginger lily, roses, hydrangeas) with a pond and island in the center.	A forest, in a valley between snowcapped mountains, composed of coniferous and deciduous trees with some sunny open lavender meadows.	A tropical beach near sunset with gentle ocean waves, large rock formations, and a palm tree jungle providing shady areas along the shore.
Interactive Items	Stones, sunflowers	Pinecones, mushrooms	Starfish, coconuts
Sounds Included	Birds (e.g., cardinal, robin), frogs, wind, rustling leaves	Birds (e.g., finch, owl, woodpecker), insects (e.g., katydid, cricket), pond trickling, wind, rustling leaves	Birds (e.g., sandpiper, seagulls), insects (e.g., cicadas, crickets) ocean waves, wind, rustling grass
Audio RMS Energy	0.014	0.007	0.033
BER: Low (0–250 Hz)	0.01	0.35	0.9
BER: Mid (250–4,000 Hz)	0.92	0.18	0.07
BER: High (4000–8,000 Hz)	0.06	0.47	0.02
Aquatic elements	Pond	Large pond	Ocean
Human-made elements	Benches, fences, bridge, path, and gazebo	Path and fences	Beach chair and umbrella
Scents	Fresh grass, rosemary, roses, blossom, wet ground	Fresh grass, lavender, pine, wet ground, woods	Beach, fresh grass, wet ground

Scents were only available for those in the OVR group. RMS, root mean square; BER, band energy ratio.

*Olfactory augmentation*: for participants in the OVR group, olfactory stimuli were delivered using a Compact Scent Generator (Olorama Technology Ltd., Valencia, Spain), placed 1–2 meters from the subject, following methods from a previous study (Abbott and Diaz-Artiles, 2022). Scent delivery was synchronized with the virtual environment using location-based triggers. When participants entered predefined regions within the environment, corresponding scents were automatically released (e.g., wet ground near water features).

If participants remained within a given region, scents were re-administered at 2-min intervals. Each scent release consisted of a 300 ms activation of the scent cartridge followed by a 9 s fan-driven dispersion phase. This pulsed approach allows for rapid onset and clearance of olfactory stimuli, supporting dynamic multisensory experiences without saturation or cross-contamination between consecutive scents. The specific scents available for each environment are detailed in Table 1.

### 2.5 Procedure

**Figure 2:**
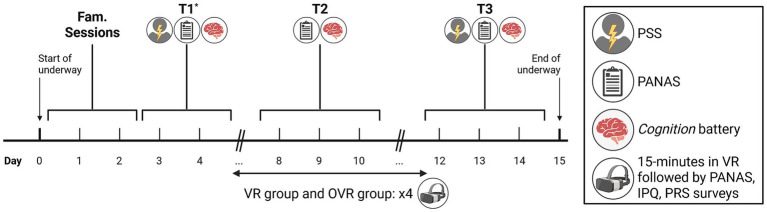
Experimental procedure overview. All participants completed a familiarization session followed by data collection points (T1, T2, T3) conducted over a 2 week period. All participants completed the Brief Resilience Scale (BRS) at T1. Participants in the VR and OVR groups were asked to complete 4 VR sessions between T1 and T3. PANAS, Positive and Negative Affect Schedule; PSS, Perceived Stress Scale; PRS, Perceived Restorativeness Scale; IPQ, iGroup Presence Questionnaire. Created in BioRender (Abbott, 2026).

The study consisted of four key time points: a familiarization session, followed by baseline (T1), mid-point (T2), and end-point (T3) assessments. The familiarization session occurred prior to baseline and was used to train participants on study procedures and cognitive tasks. Baseline assessments (T1) were conducted at least 1 day after familiarization to establish participant baselines before exposure to the VR intervention. Follow-up assessments were conducted approximately 1 week (T2) and 2 weeks (T3) after T1 to evaluate psychological and cognitive performance changes over time and potential retention of the VR intervention’s effects. All VR sessions occurred between T1 and T3. A summary of the study procedure is illustrated in Figure 2.

During the familiarization session, participants completed demographic and health surveys and practiced with the *Cognition* test battery. *Cognition* battery familiarization followed established procedures used to minimize learning effects (Basner et al., 2020). Participants received a standardized instructional presentation and video followed by administration of the cognitive battery. A shortened practice version of 8 of the 10 *Cognition* tests (no practice versions for the Visual Object Learning Test and the Balloon Analog Risk Test) was required before the first administration of each task. Practice versions remained available for subsequent sessions but were not required after familiarization. Data from this session was not included in the analysis.

T1 data collection occurred at least 1 day after the familiarization session. At T1, participants completed the *Cognition* battery and the following surveys: Positive and Negative Affect Schedule (PANAS), Brief Resilience Scale (BRS), and Perceived Stress Scale (PSS). Participants in the VR and OVR groups were also trained on the VR equipment and controls. Follow-up assessments were conducted approximately 1 week (T2) and 2 weeks (T3) after T1. At T2, participants completed the *Cognition* battery and PANAS. At T3, participants completed the *Cognition* battery, PANAS, PSS, and an exit survey to provide overall feedback. Between T1 and T3, participants in the VR and OVR groups were asked to complete four VR sessions (the control group did not receive any intervention or participate in post-intervention data collection).

### 2.6 Outcomes

Self-report surveys were administered to capture participants’ psychological mood and VR experience throughout the experiment. Additionally, a cognitive battery was administered to evaluate cognitive performance over time.

#### 2.6.1 Psychological measures

*Positive and negative affect schedule (PANAS)*: the PANAS was used to measure participants’ affective states (Watson et al., 1988), a primary outcome reflecting emotional responses to restorative environments and commonly used in VR and nature exposure research. The PANAS consists of 20 emotion-related adjectives, evenly divided into two subscales: Positive Affect (PA) and Negative Affect (NA). Positive Affect items (e.g., interested, excited, strong) capture positive and pleasant emotional states. Negative Affect items (e.g., distressed, upset, nervous) capture subjective distress and unpleasant emotional states. Participants rated the extent to which they experienced each emotion using a 5-point Likert scale (1 = Very slightly or not at all, 2 = A little, 3 = Moderately, 4 = Quite a bit, 5 = Extremely). Subscale scores were calculated as the sum of the 10 PA items and 10 NA items, respectively. Subscale scores ranged from 10 to 50, with higher scores indicating greater positive or negative affect, respectively. The PANAS internal reliability (McDonald’s omega) was *ω* = 0.89 for PA and ω = 0.77 for NA.

*Perceived stress scale (PSS)*: A validated and reliable 4-item version of the PSS was used to assess stress levels (Cohen et al., 1983), relevant to operational military settings where chronic stress exposure is common. Participants were presented with 4 questions asking how often they felt or thought a certain way in the past month and responded using a 5-point Likert scale (0 = Never, 1 = Almost never, 2 = Sometimes, 3 = Fairly often, 4 = Very often). A total PSS score (range 0 to16) was calculated as the sum of all items (items 2 and 3 reverse scored), with a higher score indicating higher levels of stress. The internal reliability (Cronbach’s alpha) was *α* = 0.73 for total PSS.

*Brief resilience scale (BRS)*: the BRS was used to evaluate participants’ resilience or the ability to recover from stress (Smith et al., 2008), as individual differences in resilience may influence responses to stress and the effectiveness of restorative interventions. Participants responded to 6 different statements using a 5-point Likert scale (1 = Strongly disagree, 2 = Disagree, 3 = Neutral, 4 = Agree, 5 = Strongly agree). A total BRS score (range 1–5) was calculated as the average of all items (items 2, 4, and 6 reverse scored), with higher values indicating greater resilience. The internal reliability (Cronbach’s alpha) was α = 0.82.

#### 2.6.2 VR experiences measures

*iGroup presence questionnaire (IPQ)*: presence is a critical component of effective VR interventions, reflecting the user’s subjective sense of “being there” in the VR environment (Slater, 2003; Slater et al., 1997). The IPQ (Schubert, 2003; Schubert et al., 2001; Regenbrecht and Schubert, 2002) consists of 14 items rated on a 7-point Likert scale, and items 2, 11, and 13 were reverse scored. Items contribute to three multi-item subscales (Experienced Realism, Involvement, and Spatial Presence) and a single-item General Presence measure (item 8). Subscale scores were computed as the means of their respective items, and Total Presence was calculated as the mean of all items. Subscale and total scores ranged from −3 to 3, with higher values indicating greater perceived presence. Internal reliability (McDonald’s omega) was *ω* = 0.64 for Experienced Realism, ω = 0.65 for Involvement, and ω = 0.67 for Spatial Presence. Consistent with previous research, scores were classified as follows: *exceptional* (above 95th percentile), *very high* (above 90th percentile), *high* (above 75th percentile), *moderate* (above 50th percentile), and *low* (below the 50^th^ percentile) (Quang Tran et al., 2024).

*Perceived Restorativeness Scale (PRS)*: In environmental psychology, restorativeness refers to the process of recovering cognitive and psychological resources that have been depleted by the demands of daily life (Hartig, 2004). The 16-item PRS (Hartig et al., 1997) was used to assess restorativeness of the virtual nature environments as it directly operationalizes constructs from Attention Restoration Theory (i.e., Being Away, Coherence, Compatibility, and Fascination). Participants indicated the extent to which a given statement describes their most recent VR experience on a 7-point Likert scale (0 = Not at all, 6 = Completely) and Coherence items were reverse scored. Subscale scores were calculated as the average of their respective items, and Total Restorativeness was calculated as the means of all items. Subscale and total scores range from 0 to 6, with higher scores indicating greater perceived restorativeness. Internal reliability (McDonald’s omega) was ω = 0.68 for Being Away, ω = 0.86 for Fascination, ω = 0.59 for Coherence, and ω = 0.92 for Compatibility.

#### 2.6.3 Cognitive performance

The *Cognition* test battery was selected due to its sensitivity to subtle performance changes in high-functioning populations and its prior use in spaceflight and operational environments (Basner et al., 2015; Moore et al., 2017). The *Cognition* battery allowed for repeated and valid assessments of cognitive performance over time and represents an essential tool for examining how human cognitive systems function and adapt under stress and fatigue. Each test systematically varied stimuli to mitigate learning effects, preserving the psychometric integrity of the assessments across repeated administrations. It includes 10 tasks evaluating a broad range of cognitive domains. The 10 tasks are the Motor Praxis (MP) task, Visual Object Learning Test (VOLT), Fractal 2-Back (F2B), Abstract Matching (AM) test, Line Orientation Test (LOT), Emotion Recognition Task (ERT), Matrix Reasoning Test (MRT), Digit Symbol Substitution Task (DSST), Balloon Analog Risk Test (BART), and Psychomotor Vigilance Test (PVT) and are briefly described in Supplementary Table 1.

*Cognition* (version 3.0.9 with the 3-min Psychomotor Vigilance Test) was administered on a laptop with the Joggle Research software (Pulsar Informatics Inc., Philadelphia, USA). Performance on all tasks is measured through two metrics: an accuracy metric and a timing (speed) metric. Accuracy outcomes spanned a range of 0–100%, with 100% signifying optimal performance. Conversely, lower values in timing outcomes denote shorter response times, indicating higher speed. Speed and accuracy metrics were adjusted for both stimulus set effects and practice effects using a short administration interval using methods described in previous research (average time between administration was 5.5 days) (Basner et al., 2020).

For most tasks, accuracy was defined as the proportion of correct responses, and the timing metric is reported as the mean reaction time over the task duration. Accuracy was not calculated for the Motor Praxis (MP) task because the instructions for this task did not emphasize accuracy. For the Fractal 2-Back (F2B) cognitive test, accuracy was calculated as the average proportion of correctly identified targets and the proportion of correctly identified decoys (correct non-responses). In the Line Orientation Test (LOT), accuracy was calculated as 3 minus the average number of clicks off, divided by 3. If the average number of clicks off was >3, then the accuracy score was set to 0. In the Balloon Analog Risk Test (BART), the number of pumps was divided by the total number of possible pumps across all 30 balloons to calculate a Risk Score. The timing metric for the PVT was calculated as 10 minus the average reciprocal reaction time (1/[Reaction time in seconds]) across the task, as this has been shown to have greater sensitivity compared to standard reaction time (Basner and Dinges, 2011). Accuracy in the PVT was calculated as:


$$\mathrm{PVT}\;\text{accuracy}=1-\frac{N\;\text{lapses}+N\;\text{false starts}}{N\;\text{stimuli}+N\;\text{false starts}}$$

Where responses ≤ 100 ms were labelled as false starts and responses ≥ 355 ms were labelled as lapses.

#### 2.6.4 Exit survey

At the conclusion of the experiment, all participants completed an exit survey consisting of open-ended questions regarding their experience. Participants were asked to describe the most challenging aspects of being underway, how they typically manage stress, and which preferred coping strategies were unavailable while underway. Participants in the VR and OVR groups were additionally asked about their perceptions of the intervention, including whether they would use it in the future, whether they found it helpful, and their preferred and least preferred virtual environments. Participants in the OVR group were further asked whether the olfactory stimuli enhanced their experience and whether any scents were perceived negatively.

### 2.7 Statistical analysis

Analyses were conducted to evaluate both immediate (post-VR) and longitudinal effects of the intervention. First, data were reviewed for quality. Three individual *Cognition* tasks were excluded from three different participants because the participants did not follow instructions. Surveys showing careless responding (e.g., selecting the same answer for all questions) were also removed. Specifically, surveys from T2, T3, and four VR sessions from one VR participant and two surveys (IPQ and PRS from a single VR session) from one OVR participant were excluded due to evidence of careless responding.

#### 2.7.1 Immediate effects of VR intervention

PANAS scores post-VR for each of the four VR sessions were initially analyzed using a repeated measures ANCOVA to determine if there was any temporal effect of the VR intervention. T1 (i.e., baseline) PANAS was used as a covariate and VR Session Number (1, 2, 3 or 4) and Group (VR or OVR) were used as fixed factors. VR Session Number was not significant, and so PANAS scores at T1 and post-VR (averaged across VR sessions for each participant) were compared using paired *t*-tests. In two OVR sessions, the scent device malfunctioned and did not release any odors; however, including or excluding these sessions did not affect conclusions from the statistical results, so they were retained in the analysis. A comparison of the statistical results with and without these sessions is provided in Supplementary Table 2. The paired differences (Post VR − T1) in PANAS PA were not normal for the VR group. However, given the small sample size, *n =* 4, the descriptive statistics (mean ± SD) of PANAS PA for the VR group are reported.

IPQ and PRS scores collected after each VR session for the VR and OVR groups were averaged by participant, and both groups (VR vs. OVR) were compared using two sample *t*-tests. Normality was assessed using the Shapiro–Wilk test and homogeneity of variance was assessed using Levene’s test. IPQ Experienced Realism scores were not normally distributed, and so a non-parametric Mann–Whitney U test was used to analyze the data. Variances were equal for all IPQ and PRS measures except for Total Presence. Standardized effect sizes are reported as Cohen’s d (or r for non-parametric tests).

#### 2.7.2 Longitudinal changes

A series of linear mixed models (LMMs) were used to assess the effect of *Group* (Control, VR, and OVR), *Time,* and *Resilience* (BRS) on longitudinal psychological and cognitive outcomes. Model fit was assessed using tests for dispersion, outliers, and distribution (i.e., Kolmogorov–Smirnov), and residual heteroscedasticity was assessed using Levene’s test. When significant assumption violations were detected, the data were fit with robust linear mixed models (RLMMS), which are robust to violations of normality and missing data (Koller, 2016). Data from one participant in the Control group and three participants in the VR group that did not complete the entire protocol (i.e., only completed T1 data collection) were included in the robust LMM analysis to utilize all data available.

Fixed factors included *Group* (Control, VR, or OVR), *Time* (measured as days since the start of the underway), and their interaction (*Group* × *Time*). Baseline *Resilience* (BRS) and the interaction *Group × Resilience* were also included to control for individual resilience and determine if the impact of the VR intervention differed based on baseline resilience. The *Resilience* and *Group × Resilience* terms were removed from the model if they were not statistically significant. Overall significance of the main and interaction effects for each model were assessed using an omnibus F-test with the Kenward-Rogers approximation for degrees of freedom. An asymptotic Wald *χ*^2^ test was applied for RLMMs because the Kenward–Rogers approximation is not supported for robust models. When the *Group* factor was significant, *post hoc* contrasts were conducted on the estimated marginal means for each *Group* averaged over *Time*. To evaluate the overall effect of *Time* across groups, the marginal trend (average slope across the three levels of *Group*) for *Time* was estimated using marginal trend analyses. When either the *Time* factor or the *Group × Time* interaction was significant, a follow-up simple slopes analysis was implemented to calculate estimated marginal slopes for each group and to test whether these slopes differed significantly from zero. If any slopes were significantly different from zero, pairwise comparisons of slopes were performed to determine whether any two group slopes differed significantly. Similarly, if *Group × Resilience* was significant, a simple slope analysis was performed. If only *Resilience* was significant, then the estimated marginal trend (averaged across groups) is reported.

The Benjamini and Hochberg correction was applied in the immediate and longitudinal analysis to adjust *p*-values and control for false discovery rate. Significance was set at *α* = 0.05 (two sided). All statistical analyses were conducted using R version 4.4.2. LMMs and RLMMs were fit using the lme4 (Bates et al., 2015) and robustlmm (Koller, 2016) packages. Diagnostics were assessed using the lmerTest (Kuznetsova et al., 2017) and DHARMa packages (Hartig, 2016). Adjusted means, slopes, and contrasts were calculated using the emmeans package (Lenth, 2025). The original datasets analyzed for this study are publicly available, and a repository can be found on GitHub: https://github.com/BHP-Lab/Immersive-VR/tree/main/FrontPsych%20Navy%20Paper.

## 3 Results

### 3.1 Immediate effects of VR intervention

**Figure 3:**
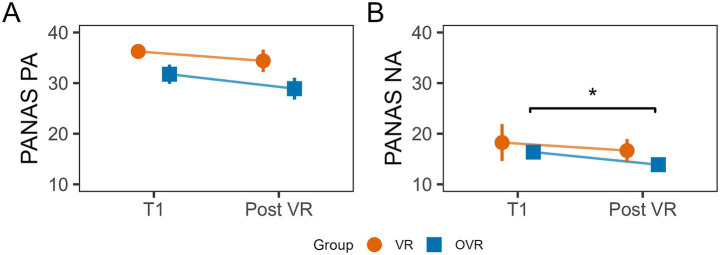
Positive affect **(A)** and negative affect **(B)** at T1 (baseline) and Post-VR for the VR group (VR, *n =* 4) and olfactory-enhanced VR group (OVR, *n =* 8). Scores may range from [10, 50]. Post-VR scores were averaged across VR sessions for each participant. Data are presented as mean ± SE. *p < 0.05.

Positive Affect (PA) and Negative Affect (NA) from the PANAS questionnaire for the VR and OVR group at T1 (baseline) and post-VR, averaged across VR sessions for each participant, are shown in Figure 3. For the VR group, PANAS PA scores were 36.3 ± 2.9 at T1 and 34.4 ± 4.4 post-VR. For the OVR group, PA scores were 31.8 ± 5.4 at T1 and 28.9 ± 6.1 post-VR, and this change was not significant [*t*(7) = −1.4, *p =* 0.3, *d =* −0.5]. For the VR group, PANAS NA scores were 18.3 ± 7.3 at T1 and 16.6 ± 4.5 post-VR, but this change was not significant [*t*(3) = −1.03, *p =* 0.38, *d =* −0.5]. For the OVR group, NA scores significantly decreased from 16.4 ± 3.7 at T1 to 13.9 ± 3.0 post-VR with a large effect size [*t*(7) = −3.7, *p =* 0.02, *d =* -1.3].

**Figure 4:**
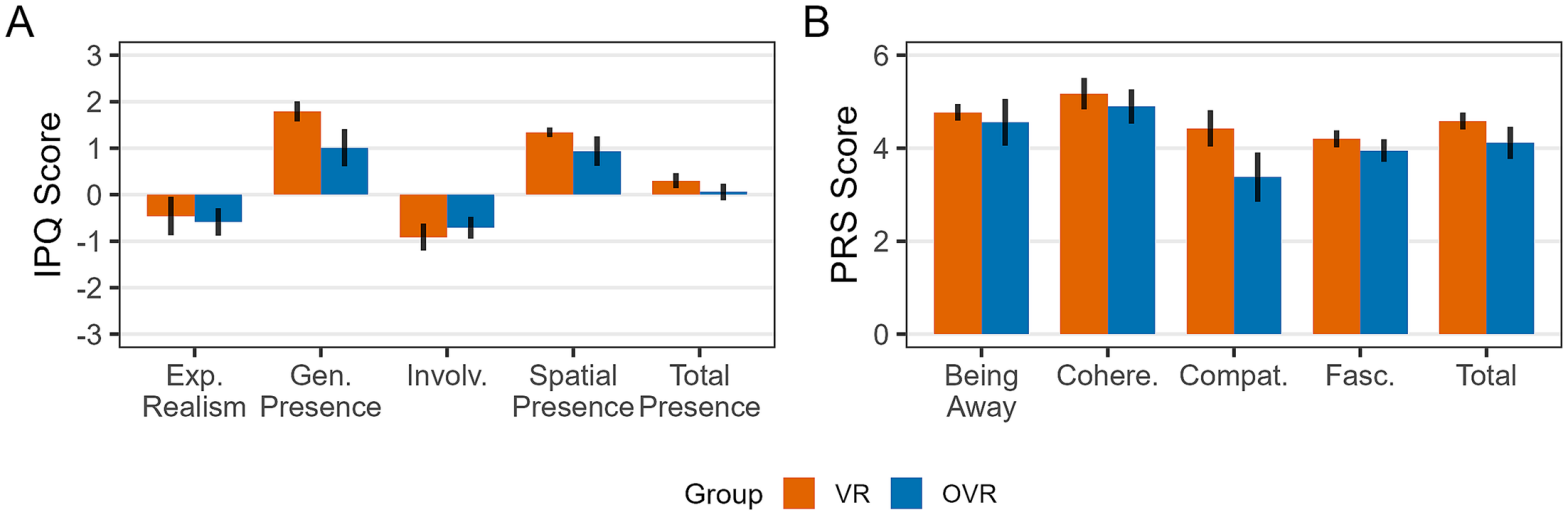
Presence and restorativeness post-VR for the VR group (VR, *n =* 4) and olfactory-enhanced VR group (OVR, *n =* 8). **(A)** iGroup Presence Questionnaire (IPQ) survey scores for the four subdomains of presence and total presence in the virtual environments. **(B)** Perceived Restorativeness Scale (PRS) survey scores for the four subdomains of restorativeness and total restorativeness of the virtual environments. Post-VR scores were averaged across VR sessions for each participant. Data are presented as mean ± SE.

IPQ presence scores and PRS scores are summarized in Figure 4. Average IPQ Experienced Realism, Involvement, and Total Presence scores were classified *low* (below the 50th percentile) for both the VR and OVR groups. The Involvement subscale relates to awareness and attention, and low scores indicate greater reported awareness of the real-world environment. Average IPQ General Presence scores were classified as *high* (above the 75th percentile) for the VR group and *moderate* (above the 50^th^ percentile) for the OVR group. Average IPQ Spatial Presence scores were *high* (above the 75^th^ percentile) for the VR group and *moderate* (above the 50^th^ percentile) for the OVR group. Further data exploration showed that low Total Presence scores were primarily driven by items 1, 7, 11, and 12, which all had average scores < −1. There was no significant difference between the VR and OVR groups for any of the IPQ subscales: Experienced Realism (W=18.0, *p =* 0.8, *r* = 0.1), General Presence [*t*(10) = 1.3, *p =* 0.68, *d =* 0.9], Involvement [*t*(10) = −0.5, *p =* 0.78, *d =* −0.3], Spatial Presence [*t*(10) = 0.9, *p =* 0.68, *d =* 0.6], or Total Presence [*t*(9.0) = 1.0, *p =* 0.68, *d =* 0.6].

Similarly, there was no significant difference between the VR and OVR groups for the PRS subscales Being Away [*t*(10) = 0.3, *p =* 0.78, *d =* 0.2], Coherence [*t*(10) = 0.5, *p =* 0.78, *d =* 0.3], Compatibility [*t*(10) = 1.2, *p =* 0.78, *d =* 0.9], Fascination [*t*(10) = 0.7, *p =* 0.78, *d =* 0.4], or Total Restorativeness [*t*(10) = 0.9, *p =* 0.78, *d =* 0.6.]

### 3.2 Longitudinal psychological state

**Figure 5:**
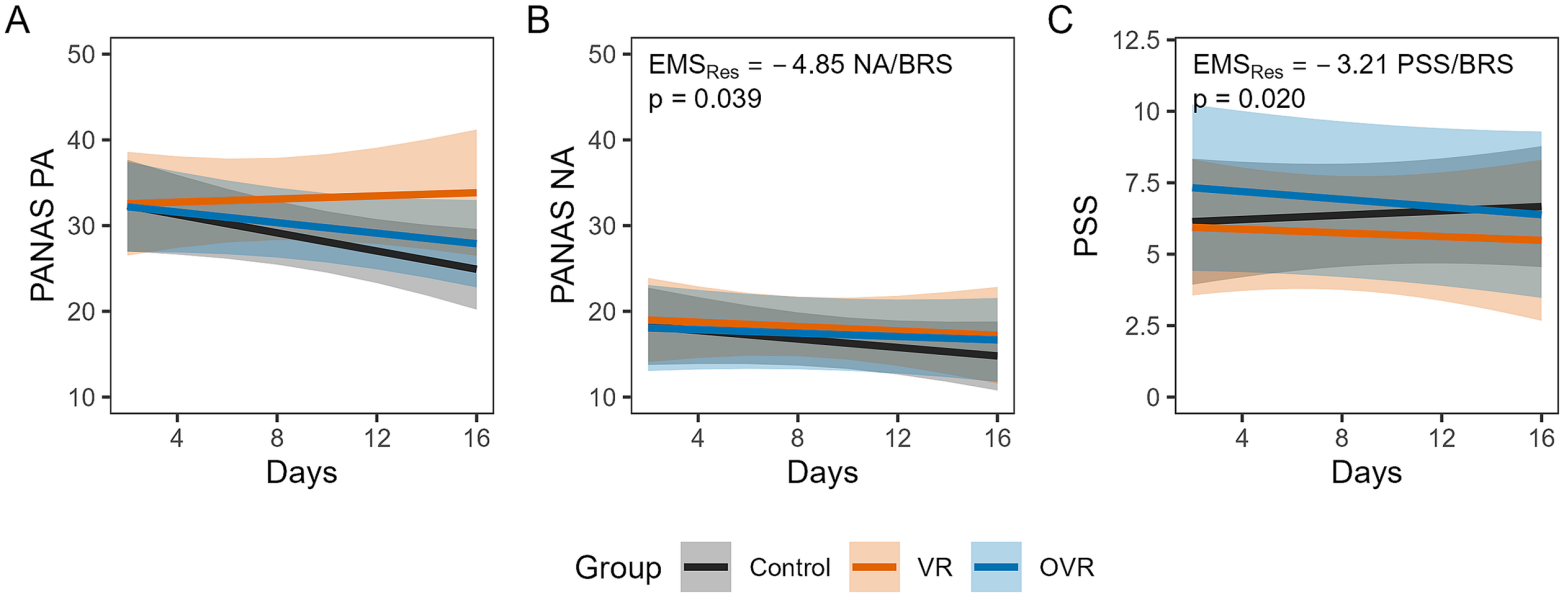
Linear mixed models for positive affect **(A)**, negative affect **(B)**, stress **(C)**. Models are presented as predicted mean ± 95% confidence interval. Fixed factors may include *Time*, *Group* (categorical: Control, VR, OVR), and *Group × Time*. NA and PSS models included the fixed factors *Resilience* and *Group × Resilience*. Significant estimated marginal slopes (EMS) are annotated within panels. EMS_Res_ denotes change in outcome per one-point increase in resilience (BRS). Subjects were included as random factors. PA, Positive Affect; NA, Negative Affect; PSS, Perceived Stress Scale; BRS, Brief Resilience Scale.

The evolution of affect (PANAS) and stress (PSS) over time for each group is shown in Figure 5. Table 2 reports the results of the LMM main effect analyses. There were no significant interaction effects between *Group* and *Time* in any of the models (all *p* > 0.16). Averaged across groups, PA (−0.249, 95% CI [−0.578, 0.081], *p =* 0.14), NA (−0.158, 95% CI [−0.437, 0.121], *p =* 0.26), and PSS (−0.021, 95% CI [−0.139, 0.097], *p =* 0.71) were stable over time.

**Table 2:** Summary of statistical results for PANAS positive affect, PANAS negative affect, and perceived stress scale (PSS) linear mixed models.

Variable	*p*-value				
	Group	Time	Group × Time	Resilience	Group × Resilience
PANAS PA	0.329	0.135	0.360	−	−
PANAS NA	0.798	0.260	0.877	**0.039**	0.595
PSS	0.785	0.716	0.672	**0.020**	0.166

Fixed factors may include *Time*, *Group* (categorical: Control, VR, or OVR), the interaction *Group* × *Time*, *Resilience*, and the interaction *Group × Resilience*. Subjects were included as random factors. Overall significance of the main and interaction effects were assessed using an omnibus F-test on each model with the Kenward-Rogers approximation for degrees of freedom. Bold values indicate significance (*p <* 0.05).

For PANAS NA and PSS, *Resilience* was significant but the interaction *Group × Resilience* was not (both p > 0.16). Since removing the interaction *Group × Resilience* changed the significance of the *Resilience* term, the interaction was retained for both the PANAS NA and PSS models. Averaged across groups, NA (−4.85, 95% CI [−9.43, −0.27], *p =* 0.039) and PSS (−3.21, 95% CI [−5.87, −0.56], *p =* 0.020) decreased with *Resilience.* For PANAS PA, there was no significant effect of *Resilience* or *Group × Resilience* (both *p* > 0.5).

### 3.3 Longitudinal cognitive performance

**Figure 6:**
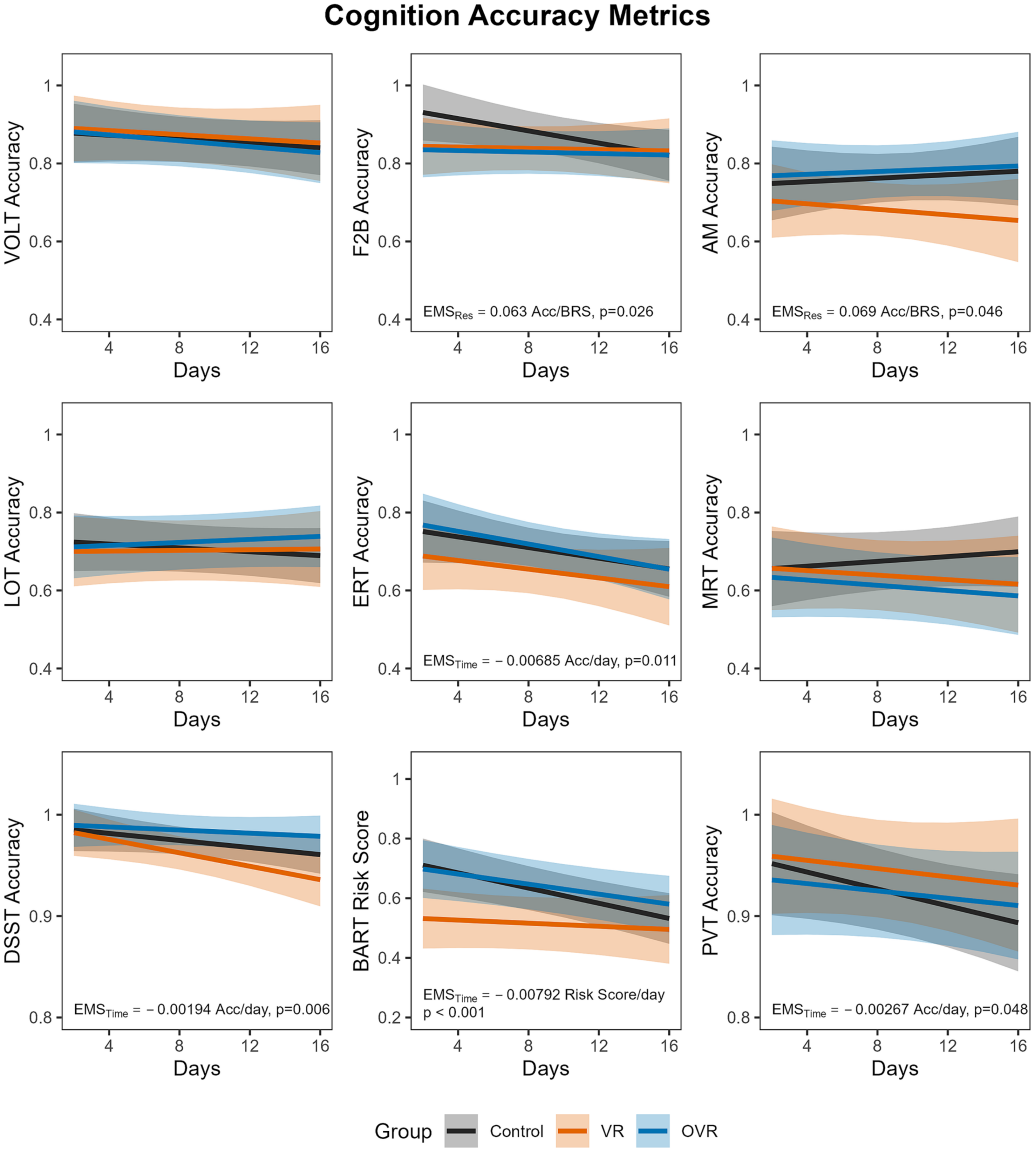
Linear and robust linear mixed models for speed metrics on the 10 tasks in the *Cognition* battery. Models are presented as predicted mean ± 95% confidence interval. Fixed factors included *Time*, *Group* (categorical: Control, VR, OVR), and *Group × Time*. The LOT model included the fixed factors *Resilience* and *Group × Resilience.* Subjects were included as random factors. Significant estimated marginal slopes (EMS) are annotated within panels. EMS_Time_ denotes change in outcome per day, and EMS_Res_ denotes change per one-point increase in resilience (BRS). Group contrasts [e.g., EMS_Time(Ctrl-OVR)_, EMS_Res(VR-OVR)_] indicate significant differential slopes between groups. VOLT, Visual Object Learning Test; F2B, Fractal 2-Back; AM, Abstract Matching; LOT, Line Orientation Test; ERT, Emotion Recognition Task; MRT, Matrix Reasoning Test; DSST, Digit Symbol Substitution Task; BART, Balloon Analog Risk Test; PVT, Psychomotor Vigilance Test; MP, Motor Praxis; BRS, Brief Resilience Scale.

The evolution of speed metrics on the 10 cognitive tasks for each group is shown in Figure 6. Table 3 reports the results of the LMM main effect analyses for both speed and accuracy metrics (measures fit with RLMMs are denoted with a^†^). There was no significant interaction observed between *Group* and *Time* for any *Cognition* metrics except for reaction time on the DSST [Wald *χ*^2^ (2) = 8.352, *p =* 0.0154]. Additionally, there was no significant main effect of *Group* observed. These results indicate that general performance and changes over time were not significantly different between groups. Additionally, there was no significant *Group × Resilience* interaction for any outcome except LOT reaction time [Wald *χ*^2^(2) = 4.1, *p =* 0.017]. The main effect of *Resilience* was significant only for reaction time on the LOT [Wald *χ*^2^(1) = 7.1, *p =* 0.008] and accuracy on the F2B [*F*(1, 23.5) = 5.7, *p =* 0.026] and AM [*F*(1, 23.7) = 4.4, *p =* 0.046].

**Table 3:** Summary of statistical results for Cognition linear and robust linear mixed models.

Variable	Units	*P-*value				
		Group	Time	Group × Time	Resilience	Group × Resilience
Speed metrics						
VOLT RT	ms	0.976	0.079	0.989	−	−
F2B RT	ms	0.816	**0.046**	0.460	−	−
AM RT	ms	0.147	**0.044**	0.581	−	−
LOT RT^†^	ms	0.070	0.940	0.997	**0.008**	**0.018**
ERT RT^†^	ms	0.432	0.320	0.473	−	−
MRT RT	ms	0.216	0.701	0.106	−	−
DSST RT^†^	ms	0.646	0.174	**0.015**	−	−
BART RT	ms	0.699	0.646	0.149	−	−
PVT Slowness^†^	10-1/s	0.8801	**<0.001**	0.859	−	−
MP RT^†^	ms	0.946	0.061	0.556	−	−
Accuracy metrics						
VOLT Accuracy	% [0–1]	0.938	0.157	0.963	−	−
F2B Accuracy	% [0–1]	0.468	0.122	0.268	**0.026**	−
AM Accuracy	% [0–1]	0.097	0.958	0.684	**0.046**	−
LOT Accuracy	% [0–1]	0.887	0.989	0.559	−	−
ERT Accuracy	% [0–1]	0.319	**0.011**	0.929	−	−
MRT Accuracy	% [0–1]	0.475	0.688	0.499	−	−
DSST Accuracy	% [0–1]	0.102	**0.006**	0.344	−	−
BART Risk Score	−	0.125	**<0.001**	0.219	−	−
PVT Accuracy^†^	% [0–1]	0.746	**0.048**	0.701	−	−

Fixed factors may include *Time*, *Group* [categorical: Control, VR, or OVR], the interaction *Group* × *Time*. *Resilience*, and the interaction *Group × Resilience*. Subjects were included as random factors. Overall significance of the main and interaction effects were assessed using an omnibus F-test with the Kenward-Rogers approximation for degrees of freedom for each model or an asymptotic Wald *χ*^2^ test for robust models. ^†^denotes robust models. VOLT, Visual Object Learning Test; F2B, Fractal 2-Back; AM, Abstract Matching; LOT, Line Orientation Test; ERT, Emotion Recognition Task; MRT, Matrix Reasoning Test; DSST, Digit Symbol Substitution Task; BART, Balloon Analog Risk Test; PVT, Psychomotor Vigilance Test; MP, Motor Praxis. Bold values indicate significance (*p <* 0.05).

Averaged over *Group*, reaction times significantly decreased over time for the F2B task (−3.13 ms/day, 95% CI [−6.2, −0.0621], *p =* 0.046) and the AM task (−22.7 ms/day, 95% CI [−44.8, −0.633], *p =* 0.044). Although the group-averaged slopes were significant, exploratory simple-slope analyses did not identify any group-specific slopes that differed significantly from zero for F2B reaction time (all *p* > 0.07) or AM reaction times (all p > 0.1). Group-averaged slope estimates for reaction time were −17.4 ms/day (95% CI [−36.8, 2.11], *p =* 0.0793) for the VOLT task and −2.94 ms/day (95% CI [−6.01, 0.138], *p =* 0.0612) for the MP task; though neither estimate differed significantly from zero.

PVT Slowness significantly increased over time (0.0192, 95% CI [0.008, 0.030], *p <* 0.001). Slowness is calculated as 10 minus the reciprocal of mean reaction time, and higher values indicate that response time increased on the PVT. Exploratory simple slopes analysis revealed that PVT Slowness significantly increased for the Control group (0.023, 95% CI [0.005, 0.040], *p =* 0.03), whereas the VR and OVR group slopes did not differ significantly from zero (VR group: 0.019, 95% CI [−0.004, 0.042], *p =* 0.11; OVR group: 0.016, 95% CI [−0.001, 0.033], *p =* 0.1). Pairwise contrasts comparing the slopes of each group indicated that there was no significant difference in slopes between groups (all *p* > 0.8).

Exploratory simple slopes analysis of DSST speed showed that reaction times increased over time for the Control group (9.481 ms/day, 95% CI [1.978, 16.984], *p =* 0.0398), whereas VR and OVR group slopes did not differ significantly from zero (VR group: 5.885 ms/day, 95% CI [−3.991, 15.760], *p =* 0.243; OVR group: −5.406 ms/day, 95% CI [−12.647, 1.834], *p =* 0.215). Furthermore, the rate of change was significantly different between the Control and OVR group (contrast = 14.9 ms/day, 95% CI [4.460, 25.314], *p =* 0.015).

Exploratory simple slopes analysis of the significant *Group × Resilience* interaction for LOT reaction time indicated that reaction times significantly increased with *Resilience* for the OVR group (5301.81 ms, 95% CI [1952.36, 8651.26], *p =* 0.006) but not the Control (258.53 ms, 95% CI [−1063.32, 1578.38], *p =* 0.98) or VR group (25.21 ms, 95% CI [−1982.26, 2032.58], *p =* 0.98). The rate of change was significantly greater for the OVR group than the Control group (contrast = −5044.28 ms, 95% CI [−8644.76, −1443.80], *p =* 0.012) and VR group (contrast = −5276.60 ms, 95% CI [−9181.57, −1371.64], *p =* 0.012).

**Figure 7:**
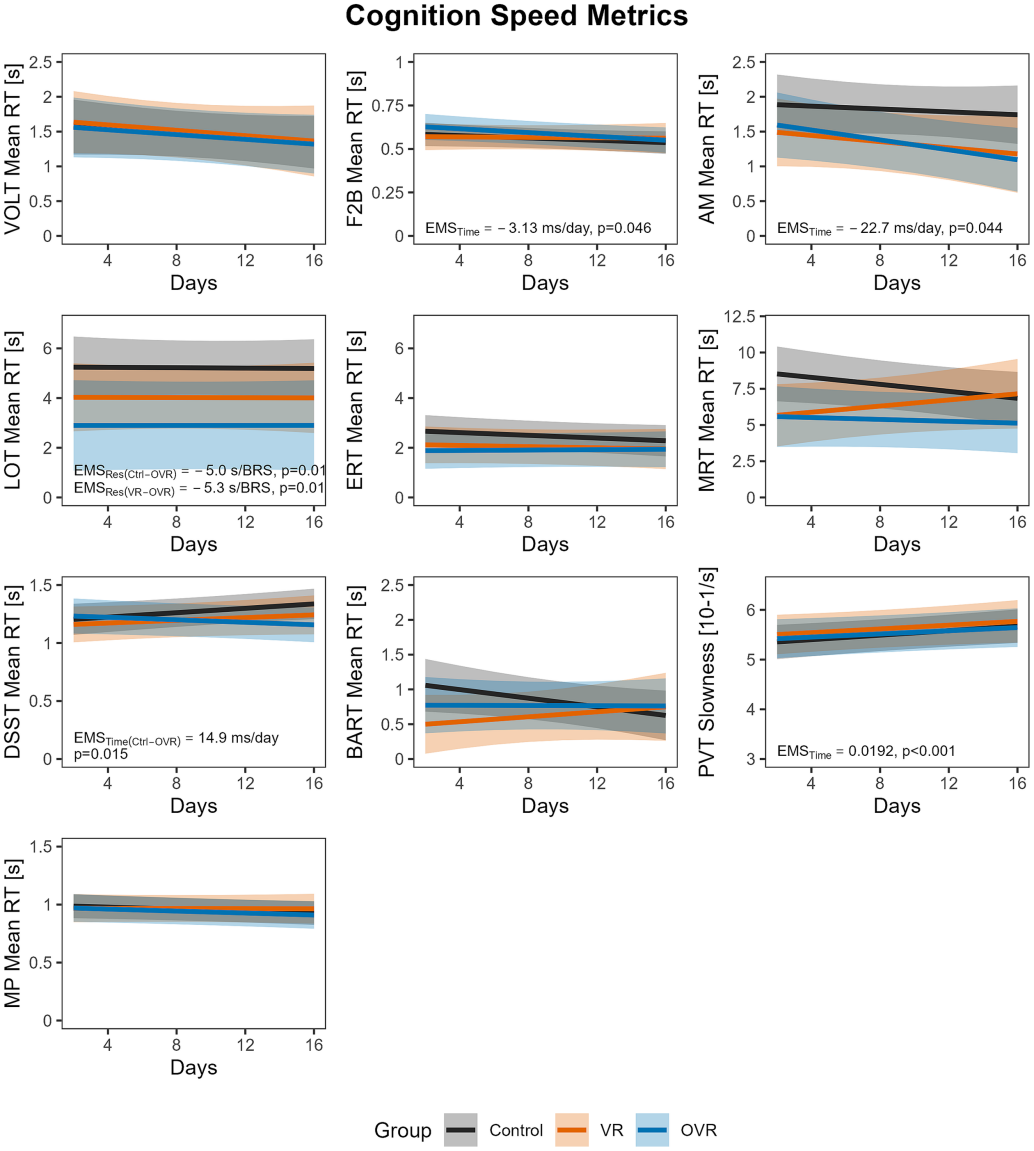
Linear and robust linear mixed models for accuracy metrics on nine tasks in the *Cognition* battery. Models are presented as mean ± 95% confidence interval. Fixed factors included *Time*, *Group* (categorical: Control, VR, OVR), and *Group × Time*. The F2B and AM models included the fixed factors *Resilience* and *Group × Resilience.* Subjects were included as random factors. Significant estimated marginal slopes (EMS) are annotated within panels. EMS_Time_ denotes change in accuracy (Acc) per day, and EMS_Res_ denotes change per one-point increase in resilience (BRS). VOLT, Visual Object Learning Test; F2B, Fractal 2-Back; AM, Abstract Matching; LOT, Line Orientation Test; ERT, Emotion Recognition Task; MRT, Matrix Reasoning Test; DSST, Digit Symbol Substitution Task; BART, Balloon Analog Risk Test; PVT, Psychomotor Vigilance Test; BRS, Brief Resilience Scale.

The evolution of accuracy metrics on nine of the cognitive tasks for each group is shown in Figure 7. Averaged over *Group*, significant reductions in accuracy over time were observed on the ERT (−0.00685, 95% CI [−0.0121, −0.00164], *p =* 0.011), DSST (−0.00194, 95% CI [−0.0033, −0.0005], *p =* 0.006), and PVT (−0.00267, 95% CI [−0.00531, −0.0000246], *p =* 0.048). Exploratory simple slopes analysis showed group-specific slopes did not significantly differ from zero for ERT accuracy (all *p* > 0.16). Exploratory simple slopes analysis for DSST accuracy showed a significant negative slope estimate for the VR group whereas the Control and OVR group slopes did not differ significantly from zero (Control: −0.002, 95% CI [−0.004, 0.000], *p =* 0.18; VR group: −0.003, 95% CI [−0.006, −0.001], *p =* 0.049; OVR group: −0.001, 95% CI [−0.003, 0.010], *p =* 0.47). Pairwise contrasts comparing the slopes of each group indicated that there was no significant difference in slopes between groups (all *p* > 0.4). Exploratory simple slopes analysis showed group-specific slopes did not significantly differ from zero for PVT accuracy (all p > 0.16). Averaged over *Group*, BART risk scores also significantly decreased over time (−0.00792, 95% CI [−0.0124, −0.0034], *p <* 0.001). Exploratory simple slopes analysis of BART risk scores showed negative slope estimates for the Control (−0.013, 95% CI [−0.020, −0.006], *p =* 0.002) and OVR group (−0.008, 95% CI [−0.015, −0.002], *p =* 0.02) whereas the VR group slope did not differ significantly from zero (−0.003, 95% CI [−0.012, 0.007], *p =* 0.5). Pairwise contrasts comparing the slopes of each group indicated that there was no significant difference in slopes between groups (all *p* > 0.25).

Additionally, accuracy on the F2B (0.063, 95% CI [0.008, 0.118], *p =* 0.026) and AM (0.069, 95% CI [0.001, 0.136], *p =* 0.046) significantly increased with *Resilience*, averaged over *Group.*

### 3.4 Qualitative feedback

Participant feedback underscored the experiential value and personal relevance of the VR intervention. All participants (11/11) in the VR groups reported that they would engage with the intervention again if it were made available, citing it as “a good way to pass the time” and “something different and interesting.” Notably, 10 out of 11 participants indicated that the VR experience was beneficial in some way, with several describing it as relaxing, emotionally restorative, or an effective psychological “escape” that facilitated a shift in mood or perspective. Across all VR sessions, the forest was selected in 24 sessions, followed by the beach and garden which were selected in 15 sessions each. The forest environment emerged as the most favored scene, selected by 7 participants, with the expansive map size and natural aesthetics cited as contributing factors; one participant specifically noted a personal affinity for woodland and mountainous landscapes. Among those in the OVR condition, 6 out of 7 participants reported that the olfactory stimuli enhanced their experience and none reported disliking any particular scent. Participants described the scents as pleasantly unexpected and memory-evoking, with one remarking that “it was a fun surprise when a scent came” and another commenting that the aromas “sparked memories.” Such responses reflect the importance of immersive, multisensory elements in shaping affective and cognitive engagement within virtual environments.

## 4 Discussion

This exploratory study characterized the psychological state and cognitive performance of sailors during a stressful underway training exercise and evaluated the immediate and longitudinal effects of a VR-based relaxation intervention. The main contributions were: (1) evaluating the feasibility and potential utility of natural VR environments for supporting behavioral health; and (2) providing a normative data set on sailor mood and cognitive performance over an extended period (~2 weeks).

Results partially supported H1. The audiovisual VR intervention alone was not associated with immediate improvements in positive or negative affect. Technical issues with the VR setup (e.g., intermittent audio and lag), discussed below, may have reduced immersion and contributed to this outcome. On the other hand, negative affect was significantly reduced in the OVR group. While preliminary and requiring replication, this pattern is consistent with the possibility that olfactory stimuli may contribute to short-term reductions in negative affect (Abbott and Diaz-Artiles, 2022), potentially through emotional and autobiographical memory pathways and reinforcing affective recovery even when overall immersion is low.

Positive affect did not increase post-VR for either group, consistent with prior laboratory (Abbott and Diaz-Artiles, 2022; Anderson et al., 2017) and analog studies (Anderson et al., 2023), although some studies have reported improvements in positive affect following virtual nature exposure (Newman et al., 2022; Valtchanov et al., 2010). This may reflect a broader pattern in which virtual nature more reliably reduces negative affect than enhances positive affect (Bolouki, 2024; Browning et al., 2020; Spano et al., 2023; Van Den Berg et al., 2003). From an Attention Restoration Theory perspective (Kaplan, 1995), simulated nature may promote disengagement from ongoing demands, allowing cognitive resources to shift away from negative mental processes, reducing negative affect without necessarily increasing positive affect. Prior work further suggests that increases in positive affect may depend on the perception of biologically relevant resources or risks within the environment, which can differ between real and simulated settings (McMahan and Estes, 2015). One possible explanation is that virtual environments may be less effective at eliciting increases in positive affect because they do not provide direct access to these resources.

Contrary to H2, the OVR group was not associated with increased presence and restorativeness compared to the VR group. Technical limitations may have degraded immersion, particularly through reduced visual fidelity (e.g., lag) and inconsistent or missing audio. Because perception of visual realism is a primary driver of presence, followed by auditory and olfactory modalities (Gonçalves et al., 2025), disruptions in visual and auditory channels may have diminished overall experience quality. Incoherent sensory inputs, including missing audio and intrusion of external sounds, may have further detracted from the virtual experience (Bessa et al., 2018; Melo et al., 2022). Additionally, operational factors, including ship motion, may have introduced vestibular-visual conflict, further reducing presence and potentially contributing to VR sickness (Gallagher and Ferrè, 2018).

Consistent with these factors, Total presence, Involvement, and Experienced Realism were classified as *low*. In particular, low scores on three of the four Involvement subscale items indicate sustained awareness of the real environment, which may be caused by technical disruptions interfering with attentional engagement. As a result, the virtual environment may not have achieved sufficient immersion and sensory coherence or simply may not have been “good enough,” to elicit strong presence (Skarbez et al., 2021). Within this context, olfactory stimuli may have had limited impact, suggesting that their effectiveness depends on a base level of immersion and coherence below which additional sensory modalities offer minimal benefit to presence and restorativeness. Another important confounding factor is that restorativeness and presence could not be directly compared across scenes. Variation in scene content, which plays a significant role in restorativeness (Shu et al., 2022; White et al., 2010), may have obscured potential effects of olfactory augmentation. On the other hand, Spatial and General Presence were classified as *moderate*-*high.* This may reflect the use of computer-generated environments with active navigation and interactive elements that supported a sense of embodiment and control in the virtual environment (Regenbrecht and Schubert, 2002).

The VR environments were generally perceived as restorative, suggesting that participants perceived some restorative qualities even under suboptimal technical conditions. It should be noted that internal reliability for the Coherence subscale was relatively low (0.59), which may obfuscate differences between groups in this subscale. Qualitative feedback further supported the perceived benefits of the VR intervention. Overall, these exploratory findings provide preliminary support of the potential psychological benefits of multisensory VR and demonstrate the feasibility of implementing such interventions in operational environments.

With respect to sustained psychological or cognitive impacts, results did not provide evidence supporting H3a or H3b in terms of consistent group-level effects over time. No significant *Group* × *Time* interactions were observed for affect, stress, or most cognitive outcomes, although the study may have been underpowered to detect small effects. Affect and stress remained stable across time for all groups. When considered alongside the observed immediate reduction in negative affect following OVR exposure, this may suggest that any affective benefits are more acute than sustained under the exposure conditions tested.

Overall, cognitive performance showed limited evidence of systematic modulation by the VR intervention. A limited number of isolated effects were observed in *post hoc* analyses (e.g., a *Group* × *Time* interaction for DSST reaction time). However, given the inconsistency of these effects across cognitive measures and sample size limitations, this finding should not be interpreted as evidence of robust intervention-related cognitive improvement. Confidence intervals for many effect estimates were wide and often encompassed effects near zero, indicating substantial uncertainty regarding both the magnitude and direction of potential intervention effects. Because acute within-session cognitive effects were not directly assessed, the present design cannot determine whether transient effects occurred immediately following VR exposure but dissipated before longitudinal measurement. Future work should examine the duration of potential psychological and cognitive effects and optimal intervention frequency.

Averaged across groups, several cognitive domains showed small but statistically significant changes over time. Small reaction time improvements (on the order of tens of milliseconds) were observed on the F2B and AM tasks, which may reflect task improvement rather than practice effects, which were controlled for during analysis. In contrast, accuracy declined in tasks involving emotion identification (ERT), complex scanning and visual tracking (DSST), and vigilant attention (PVT). Risk-taking behavior also declined over time (i.e., BART), consistent with previous findings showing reduced risk tolerance in ISS astronauts in later mission phases (Dev et al., 2024). However, these effects were small in magnitude and many had confidence intervals near zero, and results should be interpreted as exploratory observations rather than evidence of systematic cognitive decline or improvement.

Prior evidence has indicated that the amygdala and hippocampus, regions activated during the ERT and BART (Basner et al., 2015), may be particularly vulnerable to chronic stress, with downstream effects on attention, memory, and emotion processing (Lupien et al., 2009, 2018). Similarly, the PVT is minimally influenced by aptitude or practice effects (Basner and Dinges, 2011) and is highly sensitive to circadian misalignment and both immediate and chronic sleep deprivation, conditions frequently encountered in operational settings such as long-duration space missions and military deployments (Barger et al., 2014; Matsangas and Shattuck, 2021). Given these factors, future studies may benefit from examining cognitive functions related specifically to attention, memory, and emotion processing.

In the longitudinal analysis, resilience showed statistically significant associations with several psychological and cognitive outcomes. Across groups, higher baseline resilience was associated with lower negative affect and perceived stress, aligning with prior work demonstrating negative associations between resilience and negative indicators of mental health (García-León et al., 2019; Hu et al., 2015). Additionally, higher baseline resilience was associated with increased accuracy on tasks involving working memory, abstraction, and cognitive flexibility. These findings are consistent with prior work linking resilience to task and job performance (Cantante-Rodrigues et al., 2021; Yuan and Zhong, 2024), suggesting resilience acts as a protective factor buffering against the adverse effects of stress (Lara-Cabrera et al., 2021).

Although the present study did not find strong evidence of sustained cognitive benefits from OVR, the role of olfactory stimulation in supporting cognitive and psychological function merits further investigation. Prior research suggests that sensory enrichment through olfactory exposure may influence neuroplasticity-related processes, with olfactory deprivation linked to reduced neurogenesis and exposure to novel odors associated with enhanced neurogenesis (Kuhn, 2005). More recent work shows that olfactory training can increase the volume and structural connectivity of olfactory-related brain regions, which was associated with improvements in olfactory function and cognitive performance (Vance et al., 2024). While these mechanisms were not assessed in the present study, they provide a rationale for future research examining the long-term neural and cognitive effects of olfactory augmentation.

Finally, barriers to mental health care in military populations, including privacy concerns and long wait times (Kim et al., 2016; Schmied et al., 2024), highlight the need for accessible, low-burden interventions. Participants reported willingness to use the VR system, providing preliminary evidence of acceptability; however, this finding may be influenced by self-selection bias, as individuals with a prior interest in VR may have been more likely to participate. Some participants also described relying on outdoor or physical activities to manage stress, suggesting that virtual nature interventions may complement existing coping strategies. For example, pairing VR with exercise equipment has been explored in prior work (Keller et al., 2022) and may represent a feasible direction for future investigation.

### 4.1 Limitations

Several limitations should be considered. Data collection was restricted to the operational environment, and no true pre-underway baseline was available. Initial T1 assessments occurred on average 3.9 days into the underway period, potentially allowing time for participants to acclimate to operational routines. The opportunistic nature of the study also limited sample size and experimental control. Participant recruitment relied on volunteer participation, which may have introduced self-selection bias (e.g., greater enrollment among individuals interested in VR). Additionally, uncontrolled individual differences such as prior deployment experience, occupational duties, and other operational factors may have influenced the observed outcomes; therefore, group differences should not be interpreted causally More broadly, the study population included only military personnel, who undergo medical and psychological screening before service (Cardona and Ritchie, 2007). As a result, participants may differ from the general population in characteristics relevant to stress adaptation, resilience, risk tolerance, and psychological functioning. Consequently, responses to operational stressors and to interventions such as VR-based relaxation may not generalize to civilian populations or other groups with different demographic and psychological profiles.

Operational demands resulted in participant attrition and protocol deviations (e.g., multiple VR sessions per day). External factors, including simulated emergencies, may have further confounded results. The unequal distribution of participants across groups significantly limited statistical power, with attrition disproportionately impacting the VR group. Further, the small, imbalanced groups and large number of statistical tests and outcome measures assessed reduce effect size stability and increase the risk of Type I error, despite false discovery rate corrections, particularly for interaction effects and *post-hoc*/simple slope analyses. Conversely, the small sample size may have obscured effects or relationships that could have been detected with a larger sample. Accordingly, the present findings should be viewed as exploratory pending replication in larger, adequately powered samples.

Participants were allowed to select the VR scene of their choice, introducing the possibility of a habituation effect if they selected the same scene repeatedly, potentially reducing responses to the intervention. This design choice was intentional to better reflect expected operational use; however, it also reduced experimental control. Technical issues also affected the study. The original VR laptop, equipped with a more powerful GPU (NVIDIA GeForce RTX 3070 Ti), could not be used due to headset connectivity issues. A backup laptop with a weaker GPU (NVIDIA Quadro RTX 4000) was used, likely contributing to additional technical issues such as intermittent audio, noticeable lag, or both. These challenges likely compromised the quality and effectiveness of the VR intervention and may have attenuated psychological and cognitive effects. Despite these limitations, VR sickness was minimal, likely due to the inclusion of teleportation based navigation, which reduces sensory conflict and risk of VR sickness (LaViola et al., 2017; Riecke and Zielasko, 2021). This approach appeared effective: slight nausea or dizziness was reported in only five of the 45 total VR sessions.

Overall, these limitations emphasize the challenges of deploying VR in operational environments and underscore the importance of ensuring technical reliability. Future studies should incorporate larger sample sizes, pre-underway baselines, and improved technical consistency to improve generalizability and interpretability of findings.

## 5 Conclusion

This exploratory study provides preliminary evidence that olfactory-enhanced VR may have utility as an acute affective intervention, associated with short-term reductions in negative affect but not sustained psychological or cognitive benefits under the exposure conditions tested. Notably, restorativeness and presence did not differ between the audiovisual VR and OVR groups; however, only the OVR group demonstrated affective improvements. This pattern suggests that olfactory stimuli may enhance affective outcomes through mechanisms not fully captured by the presence or restorativeness measures used in this study. More broadly, this work supports the feasibility and continued investigation of multisensory virtual nature interventions as low-burden behavioral health tools in environments where access to natural settings is limited. Future research should evaluate the persistence of these effects, whether increased exposure (e.g., daily use) can support sustained psychological and cognitive benefits, assess neurophysiological correlates of emotional regulation, and expand testing to analogs such as spaceflight simulations or polar deployments.

## Data Availability

The datasets presented in this study can be found in online repositories. The names of the repository/repositories and accession number(s) can be found at: https://github.com/BHP-Lab/Immersive-VR/tree/main/FrontPsych%20Navy%20Paper Name: BHP-Lab/Immersive-VR/FrontPsych Navy Paper.

## Author’s note

Two of the authors are employees of the US Government. This work was prepared as part of our official duties. Title 17, U. S. C §105 provides that copyright protection under this title is not available for any work of the US Government. Title 17, U. S. C §101 defines a US Government work as work prepared by an employee of the US Government as part of that person’s official duties.
