# Supplementary material for: The scent of serenity: lessons learned from olfactory-enhanced virtual reality for stress reduction in isolated and confined environments
Source: Front Psychol. 2026 Jul 1;17:1769314. doi: 10.3389/fpsyg.2026.1769314 (PMC13368345; doi:10.3389/fpsyg.2026.1769314)
Supplement: Supplementary file 1 [file Table_1.DOCX]

Supplementary Material

Supplementary Table 1: Summary of the 10 cognitive tests included in the Cognition battery (Basner et al., 2015) and the cognitive domains they assess.

| **Test Name** | **Cognitive Domains Assessed** | **Description** |
| --- | --- | --- |
| 1. Motor Praxis (MP) | Sensory-motor speed | Participants clicked on squares (n = 20) that appeared randomly on the screen. The size of each subsequent square decreased. |
| 1. Visual Object Learning (VOLT) | Spatial learning and memory | Participants were asked to memorize 10 sequentially displayed 3-D figures. Next, they were asked to identify the objects they had memorized from a group of 20 similar objects (sequentially presented), half of them from the learning set and half of them new. |
| 1. Fractal 2-Back (F2B) | Working memory | Participants were presented with a series of sequential figures (fractals, n = 62) and asked to respond when the current stimulus matched the stimulus displayed two figures ago. |
| 1. Abstract Matching (AM) | Abstraction, concept formation | Subjects were presented with two pairs of objects, differing in perceptual dimensions (e.g., color and shape). Subjects were presented a target object (n = 30) they had to classify as belonging more to one of the two pairs based on a set of implicit, abstract rules. |
| 1. Line Orientation (LOT) | Spatial orientation | Participants were presented with two lines (n = 12 pairs), one stationary and one movable by clicking an arrow. Participants were asked to rotate the movable line until the perceived it to be parallel with the stationary line. |
| 1. Emotion Recognition (ERT) | Emotion identification | Participants were asked to label photographs of professional actors with different facial expressions as “happy”, “sad”, “angry”, “fearful”, or “no emotion”. There were 40 total stimuli, 8 of each emotion category. |
| 1. Matrix Reasoning (MRT) | Abstract reasoning | Participants were shown a series of patterns overlaid on a grid with one grid element missing. Subjects then selected the element that fit the pattern from a set of alternative options. There were 12 stimuli total. |
| 1. Digit Symbol Substitution (DSST) | Complex scanning and visual tracking | Participants were shown 1 of 9 symbols, each corresponding to a specific digit displayed in a legend, at a time and asked to select the correct number as quickly as possible. The test duration was fixed at 90 seconds. |
| 1. Balloon Analog Risk (BART) | Risk decision making | Participants could inflate an animated balloon or stop inflating and collect a reward proportional to the size of the balloon. If a balloon popped, no reward was granted for that stimulus. The average tendency for a set of balloons (n = 30/set) systematically differed between test administrations. |
| 1. Psychomotor Vigilance (PVT) | Vigilant attention | Subjects were asked to monitor a box on the screen and press the spacebar as quickly as possible once a millisecond counter appeared in the box. This was a 3-minute PVT with 2 to 5 second interstimulus intervals. |

Supplementary Table 2: Comparison of statistical results with and without two OVR sessions affected by scent device malfunction (no odor delivery). “Included” denotes analyses incorporating these sessions in post-VR subject-level averages, whereas “Excluded” denotes analyses with these sessions removed. Effect sizes are provided as Cohen’s d for parametric tests or r for non-parametric tests. PANAS: Positive and Negative Affect Schedule, IPQ: iGroup Presence Questionnaire, PRS: Perceived Restorativness Scale.

| **Variable** | **Test Type** | **Comparison** | **Data Inclusion** | **Statsitic** | **P-value** | **Effect size** |
| --- | --- | --- | --- | --- | --- | --- |
| PANAS Positive Affect | Paired t-test | OVR group: T1 vs. Post-VR | Included | $t(7) = -1.4$ | $0.3$ | $d=-0.5$ |
|  |  |  | Excluded | $t(7)=-1.3$ | $0.3$ | $d=-0.5$ |
| PANAS Negative Affect | Paired t-test | VR group: T1 vs. Post-VR | Included | $t(3)=-1.03$ | $0.35$ | $d=-0.5$ |
|  |  |  | Excluded | $t(3)=-1.03$ | $0.38$ | $d=-0.5$ |
| PANAS Negative Affect | Paired t-test | OVR group: T1 vs. Post-VR | Included | $t(7)=-3.7$ | $0.02$ | $d=-1.3$ |
|  |  |  | Excluded | $t(7)=-3.6$ | $0.02$ | $d=-1.3$ |
| IPQ Experienced Realism | Indep. Mann-Whitney U | VR group vs. OVR group | Included | $W=18.0$ | $0.8$ | $r=0.1$ |
|  |  |  | Excluded | $W=19.0$ | $0.67$ | $r=0.1$ |
| IPQ General Presence | Indep. t-test | VR group vs. OVR group | Included | $t(10)=1.3$ | $0.68$ | $d=0.9$ |
|  |  |  | Excluded | $t(10)=1.4$ | $0.67$ | $d=1.0$ |
| IPQ Involvement | Indep. t-test | VR group vs. OVR group | Included | $t(10)=-0.5$ | $0.78$ | $d=-0.3$ |
|  |  |  | Excluded | $t(10)=-0.5$ | $0.67$ | $d=-0.3$ |
| IPQ Spatial Presence | Indep. t-test | VR group vs. OVR group | Included | $t(10)=0.9$ | $0.68$ | $d=0.6$ |
|  |  |  | Excluded | $t(10)=0.9$ | $0.67$ | $d=0.6$ |
| IPQ Total Presence | Indep. t-test | VR group vs. OVR group | Included | $t(9)=1.0$ | $0.68$ | $d=0.6$ |
|  |  |  | Excluded | $t(10)=1.1$ | $0.67$ | $d=0.6$ |
| PRS Being Away | Indep. t-test | VR group vs. OVR group | Included | $t(10)=0.3$ | $0.78$ | $d=0.2$ |
|  |  |  | Excluded | $t(10)=0.3$ | $0.8$ | $d=0.2$ |
| PRS Coherence | Indep. t-test | VR group vs. OVR group | Included | $t(10)=0.5$ | $0.78$ | $d=0.3$ |
|  |  |  | Excluded | $t(10)=0.4$ | $0.8$ | $d=0.3$ |
| PRS Compatibility | Indep. t-test | VR group vs. OVR group | Included | $t(10)=1.2$ | $0.78$ | $d=0.9$ |
|  |  |  | Excluded | $t(10)=1.4$ | $0.6$ | $d=0.9$ |
| PRS Fascination | Indep. t-test | VR group vs. OVR group | Included | $t(10)=0.7$ | $0.78$ | $d=0.4$ |
|  |  |  | Excluded | $t(10)=0.9$ | $0.6$ | $d=0.6$ |
| PRS Total Restorativeness | Indep. t-test | VR group vs. OVR group | Included | $t(10)=0.9$ | $0.78$ | $d=0.6$ |
|  |  |  | Excluded | $t(10)=1.0$ | $0.6$ | $d=0.7$ |
